# Analysis of the impact of adherence to guidelines and expert advice in patients with myelodysplastic syndromes

**DOI:** 10.1007/s00277-020-04325-7

**Published:** 2020-11-07

**Authors:** A. Kasprzak, K. Nachtkamp, M. Kondakci, T. Schroeder, G. Kobbe, A. Kündgen, J. Kaivers, C. Rautenberg, R. Haas, N. Gattermann, N. Bonadies, U. Germing

**Affiliations:** 1 Department of Hematology, Oncology and Clinical Immunology, University Hospital Duesseldorf, Duesseldorf, Germany; 2 Department of Hematology and Central Hematology Laboratory, Inselspital, Bern University Hospital, University of Bern, Bern, Switzerland

**Keywords:** Guideline adherence, Myelodysplastic syndrome, Hematopoietic stem cell transplantation, Iron chelation therapy, Lenalidomide, Hypomethylating agents

## Abstract

The European Leukemia Net (ELN) guidelines for treatment of myelodysplastic syndromes (MDS) connect heterogeneous MDS subgroups with a number of therapeutic options ranging from best supportive care to allogeneic stem cell transplantation (alloSCT). However, it is currently unknown whether adherence to guideline recommendations translates into improved survival. The sizeable database of the Duesseldorf MDS Registry allowed us to address this question. We first performed a retrospective analysis including 1698 patients (cohort 1) to whom we retrospectively applied the ELN guidelines. We compared patients treated according to the guidelines with patients who deviated from it, either because they received a certain treatment though it was not recommended or because they did not receive that treatment despite being eligible. We also performed a prospective study with 381 patients (cohort 2) who were seen in our department and received guideline-based expert advice. Again, we compared the impact of subsequent guideline-adherent versus non-adherent treatment. For the majority of treatment options (best supportive care, lenalidomide, hypomethylating agents, low-dose chemotherapy, and intensive chemotherapy), we found that adherence to the ELN guidelines did not improve survival in cohort 1. The same was true when patient management was prospectively enhanced through guideline-based treatment advice given by MDS experts (cohort 2). The only exceptions were alloSCT and iron chelation (ICT). Patients receiving ICT and alloSCT as recommended fared significantly better than those who were eligible but received other treatment. Our analysis underscores the limited survival impact of most MDS therapies and suggests to pursue alloSCT in all suitable candidates.

**Supplementary Information:**

The online version contains supplementary material available at 10.1007/s00277-020-04325-7.

**Figure:**
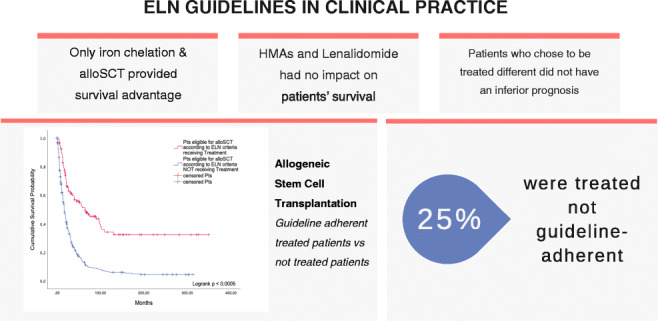
Graphical abstract

## Introduction

Myelodysplastic syndromes (MDS) are a heterogeneous group of hematological malignancies that vary considerably with regard to life expectancy. Prognosis depends on disease-related as well as patient-related factors. Prognostic scoring systems are generally built on disease-related factors and designed to separate patients into risk groups. These risk groups are utilized by guidelines to provide risk-adapted treatment recommendations within the framework of approved drugs and procedures. Guideline-based therapeutic decision-making is enhanced by expert advice that takes patient-related factors like performance status and comorbidities into account. Widely accepted MDS guidelines that connect MDS subtypes and risk groups with a number of therapeutic options are the European LeukemiaNet (ELN) [1] guidelines for adult patients with primary MDS, the National Comprehensive Cancer Network (NCCN) guidelines for MDS [2], the recommendations issued by the Nordic MDS Study Group [3], and the recommendations for allogeneic hematopoietic stem cell transplantation for MDS and chronic myelomonocytic leukemia (CMML) issued by an international expert panel [4].

However, the survival impact of adherence to MDS guidelines and guideline-based expert advice is unknown. We addressed this question by analyzing adherence to ELN guidelines and MDS expert recommendations in the setting of the large Duesseldorf MDS Registry.

## Patients and methods

### Cohort 1

Our retrospective analysis included 1659 patients in the Duesseldorf MDS Registry who were diagnosed between 1982 and 2014 (cohort 1). The main purpose of this analysis was to evaluate the survival impact of adherence to guidelines in clinical routine. As guidelines were not published until 2006, clinical decision-making in our cohort 1 could not be guideline-based in a large proportion of cases. Therefore, we retrospectively applied the current ELN guidelines to define “guide-line adherent therapy.” The following drugs and treatment modalities were evaluated: erythropoiesis stimulating agents (ESA), iron chelation therapy (ICT), red blood cell (RBC) and platelet transfusions, lenalidomide, hypomethylating agents (HMA), induction, and allogeneic stem cell transplantation (alloSCT). For each patient in cohort 1, we determined whether the received treatment was in accordance with ELN guideline recommendations. We also paid attention to drug approval status at the time of treatment and determined whether treatment was in-label or off-label. Patients who received approved treatment they were eligible for were classified as guideline-adherent and in-label. In both cohorts, overall survival was used as the primary endpoint. Overall survival of guideline-adherent and non-adherent patients was finally compared using the Kaplan-Meier method and the log rank test. The defined starting point was the time of diagnosis. Furthermore, 1146 patients (69.8%) were treated in our department, while 495 patients (30.2%) underwent treatment elsewhere. No statistical difference in median overall survival could be found between these two subgroups. Patients in this cohort were followed for a median of 22 months (1–500). Previous to comparing the subgroups in this cohort, we performed analyses to check whether there are potential confounding factors. We investigated age, IPSS, and MDS CI. Our analyses showed no significant difference between these groups, which made them comparable. This applies to all subgroups.

### Cohort 2

Subsequently, we performed a prospective analysis on 381 additional patients (cohort 2) who first presented to the MDS outpatient clinic at the University Hospital of Duesseldorf between 2015 and 2018. The objective of this analysis was to assess the survival impact of adherence to guideline-based expert advice in an outpatient setting. In order to document all first and follow-up visits in a standardized manner, we established a tool that captures prognostic scores (International Prognostic Scoring System (IPSS) [5], MDS Comorbidity Index (MDS CI) [6], and Hematopoietic Stem Cell Transplantation Comorbidity Index (HCT CI) [7]) and specific therapeutic advice given at each visit. Documented therapeutic advice included watchful waiting, best supportive care (transfusions, ICT, ESA), HMA, lenalidomide, intensive and non-intensive chemotherapy, and alloSCT. We then compared the outcome of patients who followed our therapeutic advice with patients who, for various reasons, did not. As most of the interventions take a certain time to be started, a time-dependent approach was applied: patients were classified as adherent when the recommended therapeutic measure was initiated within three months after the first visit. Potential candidates for alloSCT were considered as adherent, when alloSCT was initiated within a period of six months past their first visit in our outpatient clinic. In this cohort, 44.9% of the patients (*n* = 171) received treatment in our outpatient clinic, whereas 55.1% (*n* = 210) were treated elsewhere. Median overall survival did not differ significantly between these two groups. Patients not treated in our outpatient clinic were followed by contacting primary care physicians and hematologists. In 5.2% of the patients, a comprehensive follow-up was not possible.

Cohort 2 was analyzed using a matching procedure. The following parameters were taken into account: age (± 7 years), gender, blood cell counts, IPSS [5], and MDS CI [6]. For patients undergoing alloSCT, the HCT CI [7] was also included. Again, median overall survival was the major endpoint. Each treatment was evaluated by comparing median overall survival in the entire group of patients receiving the respective treatment with median survival in a group of matched patients from a historical cohort from the Duesseldorf MDS Registry who only received BSC. It is known that MDS patients diagnosed after 2002 show a superior overall survival compared to patients diagnosed before 2002. This effect is especially pronounced in patients with a high-risk disease, but could also be verified in patients receiving BSC only [8]. Thus, we split the matching partners in all therapeutic categories according to time of diagnosis using a cutoff at 2002 and compared their OAS beforehand, to ensure that if matching partners fared worse, it cannot be assigned to them not receiving contemporary supportive care. For matching partners in all examined therapeutic categories, we could not find a significant difference in OAS between the two groups. To enhance the analysis, each treatment group was split into lower-risk and higher-risk MDS. Lower-risk MDS included IPSS low and intermediate-1, whereas higher-risk MDS was defined by intermediate-2 and high-risk according to IPSS. Due to this approach, subanalyses were restricted to a relatively low number of patients. Patients included in this analysis were followed until July 2018, with a median follow-up time of 14 months (1–195).

## Results

### Retrospective cohort (cohort 1)

#### Erythropoiesis stimulating agents

##### Patients receiving ESA according to the ELN guidelines versus patients receiving ESA without guideline endorsement

When we looked at erythropoiesis stimulating factors, we found that 57 patients in cohort 1 received erythropoiesis stimulating agents (ESA) in-label and guideline-adherent, while six patients were treated off-label. Patients treated according to the guidelines lived for a median of 65 months (95%CI: 37.2; 97.8), while patients treated outside the guidelines lived for a median of 173 months (95%CI: 0.0; 379.4). However, the difference was statistically not significant (*p* = 0.099).

##### Patients receiving ESA according to the ELN guidelines versus patients eligible for ESA treatment but not receiving it

We identified 15 patients who did not receive ESA despite being eligible according to the guidelines. Their survival (median 29 months, 95%CI: 0.0; 70.8) was statistically not different from that of the 57 patients receiving ESA according to the guidelines (median 65 months, 95%CI: 37.2; 97.8) (*p* = 0.509).

#### Iron chelation therapy

##### Patients receiving ICT according to the ELN guidelines versus patients receiving ICT without guideline endorsement

Fifty-three patients received ICT in-label and guideline-adherent and lived for a median of 70 months (95%CI: 48; 92). In addition, 19 patients received ICT off-label and lived for a median of 90 months (95%CI: 40.1; 139.9). The difference was statistically not significant (*p* = 0.652).

##### Patients receiving ICT according to the ELN guidelines versus patients eligible for ICT but not receiving it

**Fig. 1:**
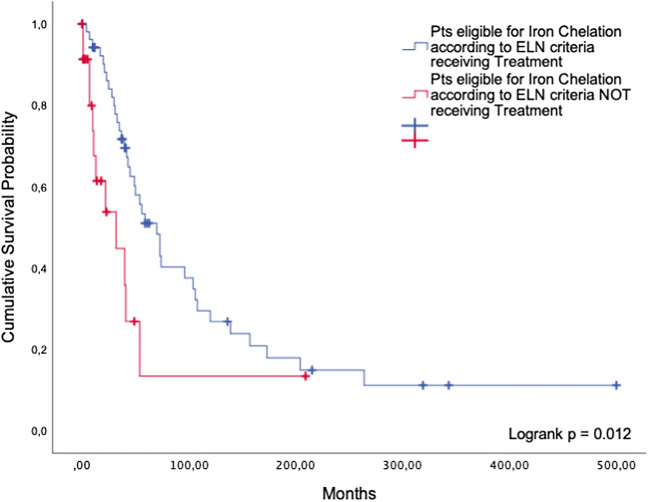
Iron chelation therapy. Comparison between guideline-adherent patients and patients eligible for but not receiving ICT

The group of 53 patients who were treated in-label and guideline-adherent with an iron chelator achieved a survival benefit compared with 24 patients who were eligible according to the ELN guidelines but never received ICT (median overall survival 70 months (95%CI: 48; 92) versus 32 months (95%CI: 2.4; 61.6)). The difference was statistically significant (*p* = 0.012, Fig. 1).

#### Lenalidomide

##### Patients receiving lenalidomide according to the ELN guidelines versus patients receiving lenalidomide without guideline endorsement

Sixteen patients received lenalidomide in accordance with the ELN guidelines, with a median overall survival of 69 months (95%CI: 47.86; 90.14). Their survival was not significantly different from 34 patients who did not meet the ELN criteria but were still treated with lenalidomide (median 77 months, 95%CI: 34; 120) (*p* = 0.639). According to our review of documents, these patients did not meet the ELN criteria because they received lenalidomide despite not being RBC transfusion dependent.

##### Patients receiving lenalidomide according to the ELN guidelines versus patients eligible for lenalidomide treatment but not receiving it

For the 16 patients receiving lenalidomide according to the guidelines, survival was not significantly different when compared to 13 patients who received only BSC despite being eligible for lenalidomide treatment (median overall survival 65 months (95%CI: 47.9; 90.1) versus 93 months (95%CI: 0.0; 210.4)) (*p* = 0.677).

#### Hypomethylating agents

##### Patients receiving HMAs according to the ELN guidelines versus patients receiving HMAs without guideline endorsement

Among 66 patients treated with azacitidine, only one patient received it outside the guideline recommendations. We were thus not able to formally compare guideline-adherent and non-adherent treatment.

##### Patients receiving HMAs according to the ELN guidelines versus patients eligible for HMA treatment but not receiving it

**Fig. 2:**
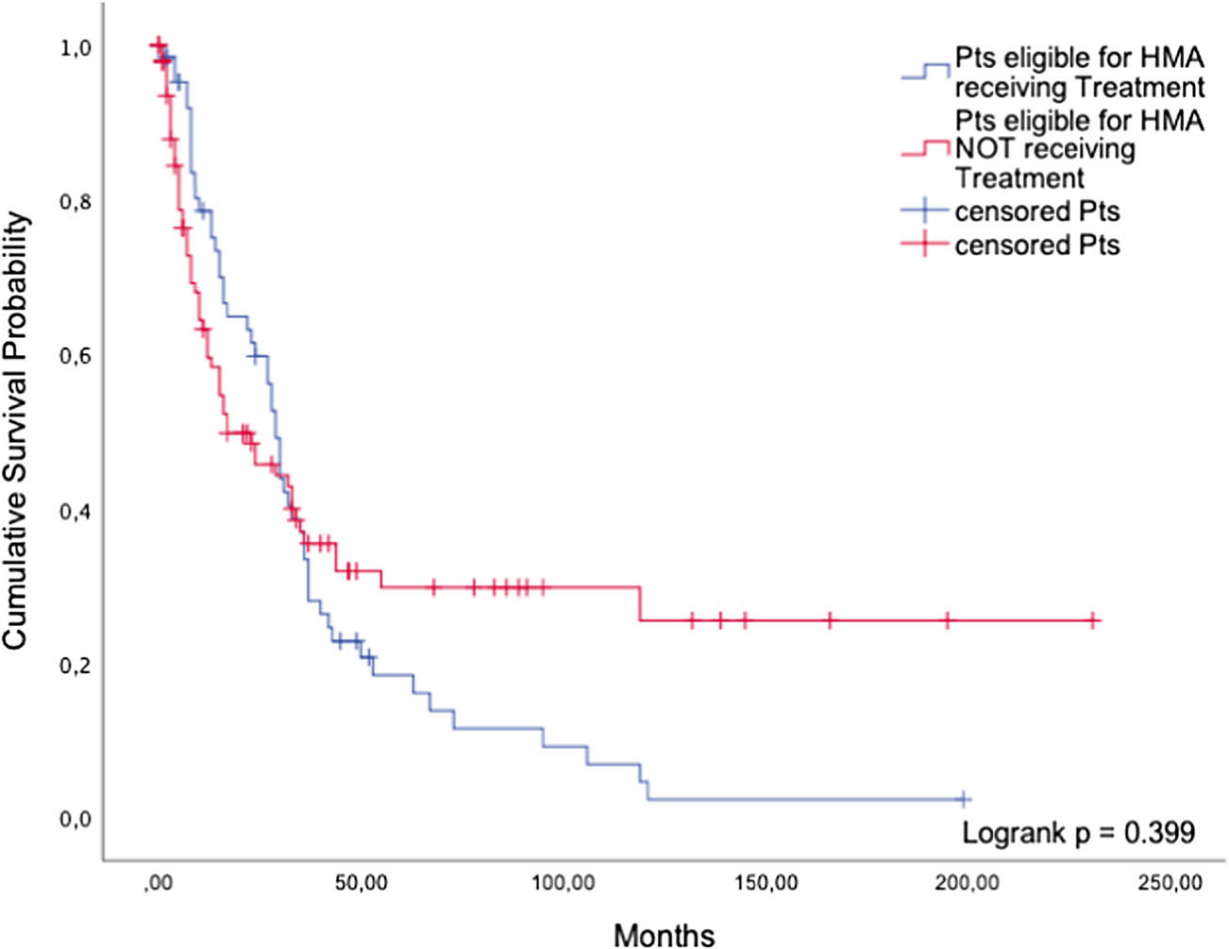
Hypomethylating agents. Comparison of guideline-adherent patients and eligible patients not receiving HMAs

Sixty-five patients were treated with azacitidine, whereas 100 patients were not treated even though they were eligible according to the guidelines. The median overall survival of treated patients was 29 months (95%CI: 25.9; 32.1) while patients who received best supportive care only lived for a median of 17 months (95%CI: 4.9; 29.1) (*p* = 0.399, Fig. 2).

#### Allogeneic stem cell transplantation

##### Patients undergoing alloSCT according to the ELN guidelines versus patients receiving alloSCT without guideline endorsement

**Fig. 3:**
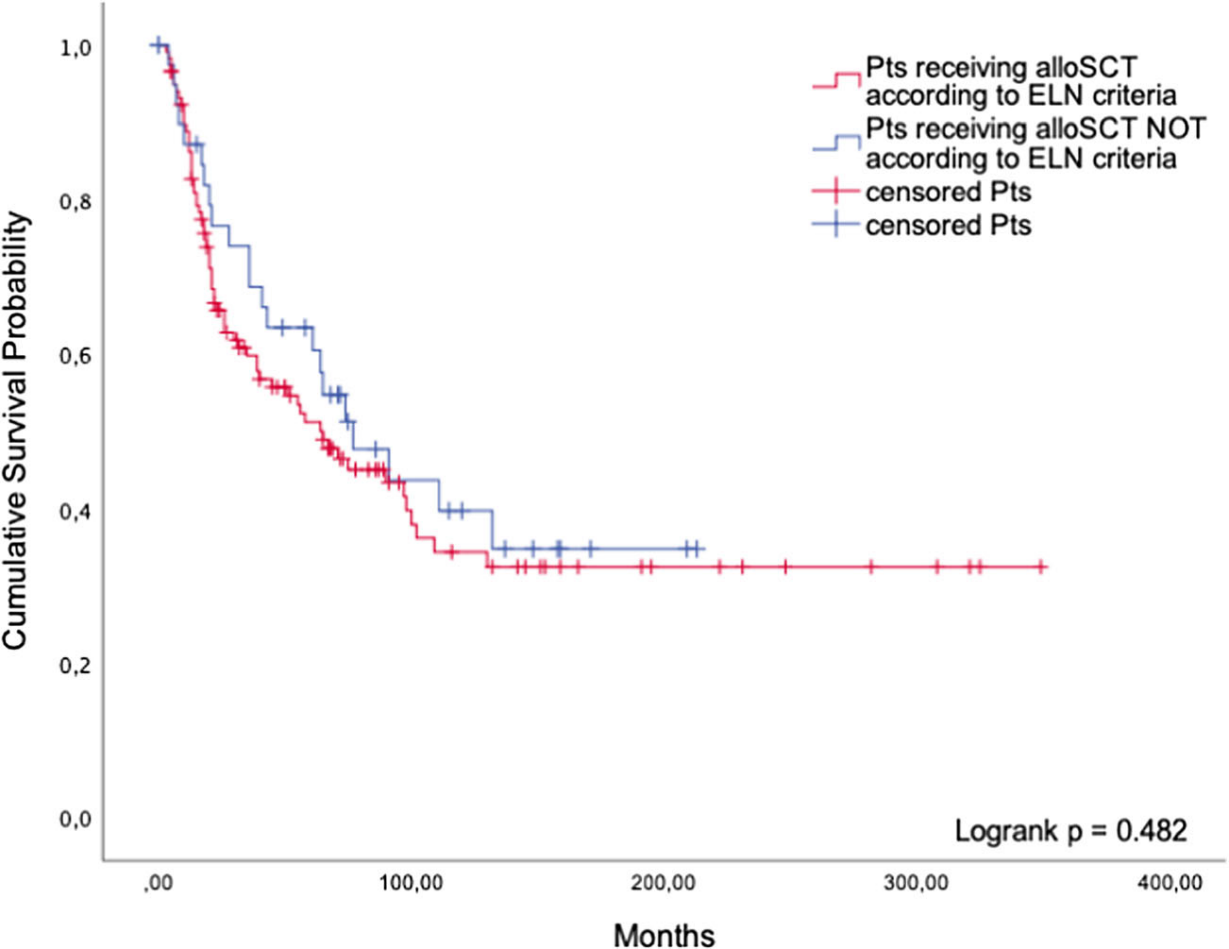
AlloSCT. Comparison of guideline-adherent patients and patients undergoing alloSCT without guideline endorsement

The aforementioned therapies are all non-intensive. In contrast, alloSCT represents the most intensive and potentially toxic but also potentially curative treatment applicable to MDS patients. Cohort 1 included 118 patients undergoing alloSCT in accordance with the ELN guidelines, whereas 40 patients did not meet the ELN guideline criteria but were still transplanted. In the latter group, 25 patients (62.5%) had only mild cytopenias and a blast percentage below the required threshold, or a favorable karyotype. Further, thirteen patients (32.5%) with a low-risk IPSS underwent alloSCT, and two patients (5.0%) were transplanted while being older than 70 years. The median survival of patients who were transplanted despite not being formally eligible was 77 months (95%CI: 44.7; 109.4), compared to 65 months in guideline-adherent patients (95%CI: 29.9; 100.1, *p* = 0.482, Fig. 3).

##### Patients undergoing alloSCT according to the ELN guidelines versus patients eligible for alloSCT but not receiving it

**Fig. 4:**
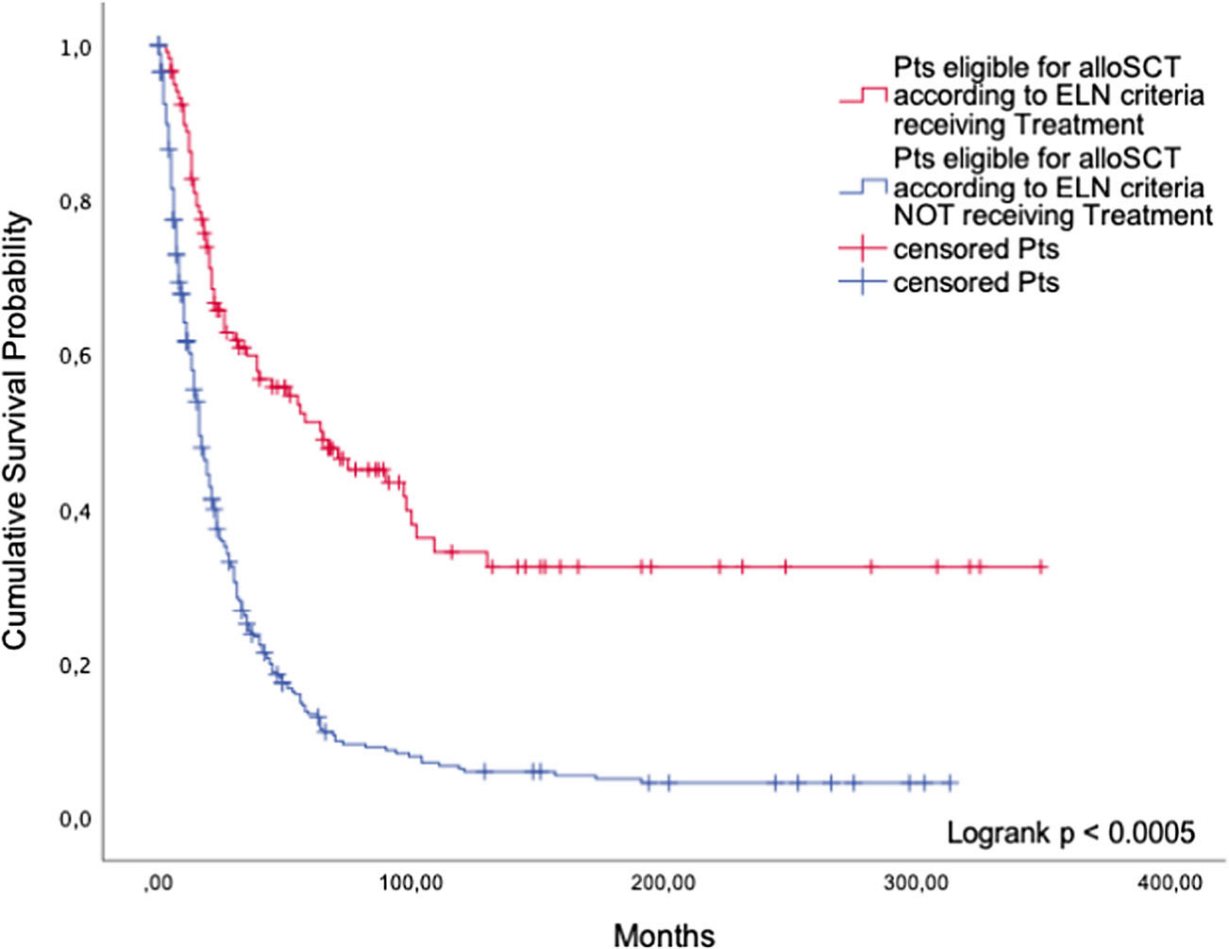
AlloSCT. Comparison of guideline-adherent patients and patients not undergoing alloSCT despite being eligible

While 118 patients underwent alloSCT in accordance with the guidelines, 348 patients were not transplanted even though they were eligible. Patients undergoing alloSCT lived significantly longer than patients who were eligible but were not transplanted (*p* < 0.0005, Fig. 4). Median survival of patients undergoing alloSCT was 65 months (95%CI: 29.9; 100.1) compared to 16 months (95%CI: 13.6; 18.5) in patients who received BSC only.

### Prospective cohort (cohort 2)

#### Watchful waiting

In cohort 2, 85 patients received the advice to watch and wait. Since they were in no need for any treatment at their first visit, they were simply asked to return for follow-up visits. Forty-nine of them adhered to this recommendation, while 31 patients decided to try a more active approach. Fourteen patients (45.2%) received BSC, and six (19.4%) were treated with HMAs. Three patients (9.7%) received lenalidomide, and another three underwent chemotherapy. Two non-adherent patients (6.5%) decided to undergo alloSCT. The alternative therapeutic approach of three patients was unknown.

The non-adherent patients appeared to live longer (median 265 months (95%CI: 194.9; 335.6)) than the adherent patients (91 months (95%CI: 67.8; 114.4)), but the difference was statistically not significant (*p* = 0.799).

#### Best supportive care

Among 100 patients recommended to restrict treatment to best supportive care, including RBC and platelet transfusions, HGFs, and ICT, 84 were adherent, whereas 13 chose to try more intensive treatment. Due to small numbers of patients in the subgroups, a separate analysis for ICT as performed in the retrospective cohort was not feasible. In this group, five patients (38.5%) decided to be treated with BSC. Four patients (30.8%) underwent alloSCT and two (15.4%) received azacitidine. Two patients (7.7%) were treated using lenalidomide. The two groups did not differ significantly in terms of overall survival (median 188 months (95%CI: 81.2; 294.8) versus 106 months (95%CI: 50.9; 161.1)) (*p* = 0.664). Matched-pair analysis was not necessary for the comparison between BSC and more intensive treatment, since all patients in this group received BSC anyway.

#### Lenalidomide

Thirteen patients were recommended to start treatment with lenalidomide, 9 of whom actually received this immunomodulatory drug while four did not. These four patients chose to receive BSC only. All patients were classified as lower-risk MDS. Since all patients were alive at the end of the study, no conclusions could be drawn as to a possible survival advantage. However, a matched-pair analysis provided valuable clues. When matching the nine adherent patients with 30 patients receiving BSC only, the adherent patients showed a significant survival benefit (median OS 182 months (95%CI: 148.7; 216.5) versus 73 months (95%CI: 40.2; 107.0)) (*p* = 0.004). Patients in both of the groups had MDS del5q as defined by the WHO.

#### Hypomethylating agents

**Fig. 5:**
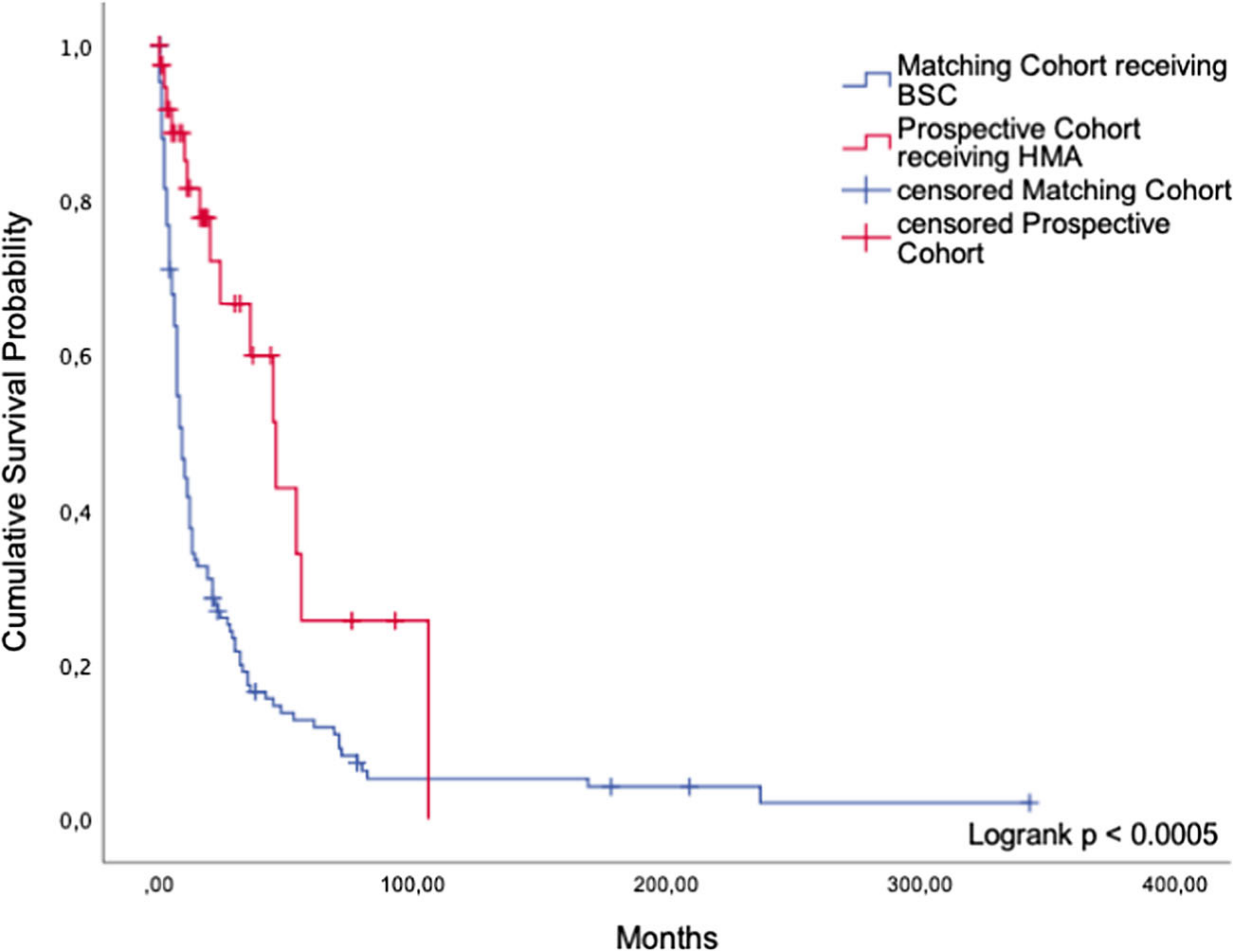
Hypomethylating agents. Comparison of cumulative survival between patients in the prospective cohort receiving HMA and patients receiving BSC only

Fifty-three patients were advised to be treated with azacitidine. Comparing 33 adherent with 15 non-adherent patients, no significant difference in survival was found (median OS 33 months (95%CI: 17.1; 58.8) versus 54 months (95%CI: 11.1; 96.6)) (*p* = 0.197). Nine patients (60%) decided to follow a best supportive care concept; three (20%) underwent chemotherapy while another two (13.3%) were transplanted. One patient (6.6%) did not receive any therapy at all. In five cases, a comprehensive follow-up was not possible. Again, the matching procedure yielded useful information and verified the trend we showed in patients receiving HMAs in the retrospective group. Thirty-three adhering patients showed a highly significant survival advantage over 128 matched patients who received BSC but would have been eligible for treatment with azacitidine as well (median OS 46 months (95%CI: 31.3; 60.7) versus 9 months (95%CI: 7.2; 10.8)) (*p* < 0.005, Fig. 5). The 128 matching partners were part of a historical control cohort. Since azacitidine was not yet approved at the time of their first diagnosis, these patients did not receive a recommendation for treatment with azacitidine.

#### Intensive and non-intensive chemotherapy

Of 16 patients who were advised to undergo chemotherapy, nine received this treatment, while the other seven patients opted for another therapeutic approach. Three patients (42.9%) received azacitidine, while two (28.6%) decided for BSC. Two other patients (28.6%) underwent alloSCT instead. Overall survival was not significantly different (median 35 months (95%CI: 23.6; 46.6) versus 32 months (95%CI: 20.6; 43.6)) (*p* = 0.954). We were not able to identify matching partners who only received BSC.

#### Allogeneic stem cell transplantation

Among 95 patients who were advised to undergo alloSCT, 63 patients were transplanted within six months, whereas 26 patients were treated differently. Nine patients (27.4%) decided for BSC, another nine (27.4%) received HMAs. Four patients (15.4%) decided for watchful waiting. Four patients (15.4%) underwent chemotherapy. Due to comorbidities, eleven non-adherent patients (42%) could not be transplanted. Seven patients (26%) declined the option of alloSCT due to various personal reasons. For five other patients (19%), we were not able to find a suitable donor within a period of six months.

Without applying our matching criteria, a survival benefit of alloSCT was noted (median OS 74 months (95%CI: 25.05;122.95) versus 28 months (95%CI:7.52; 48.48)) (*p* = 0.015).

**Fig. 6:**
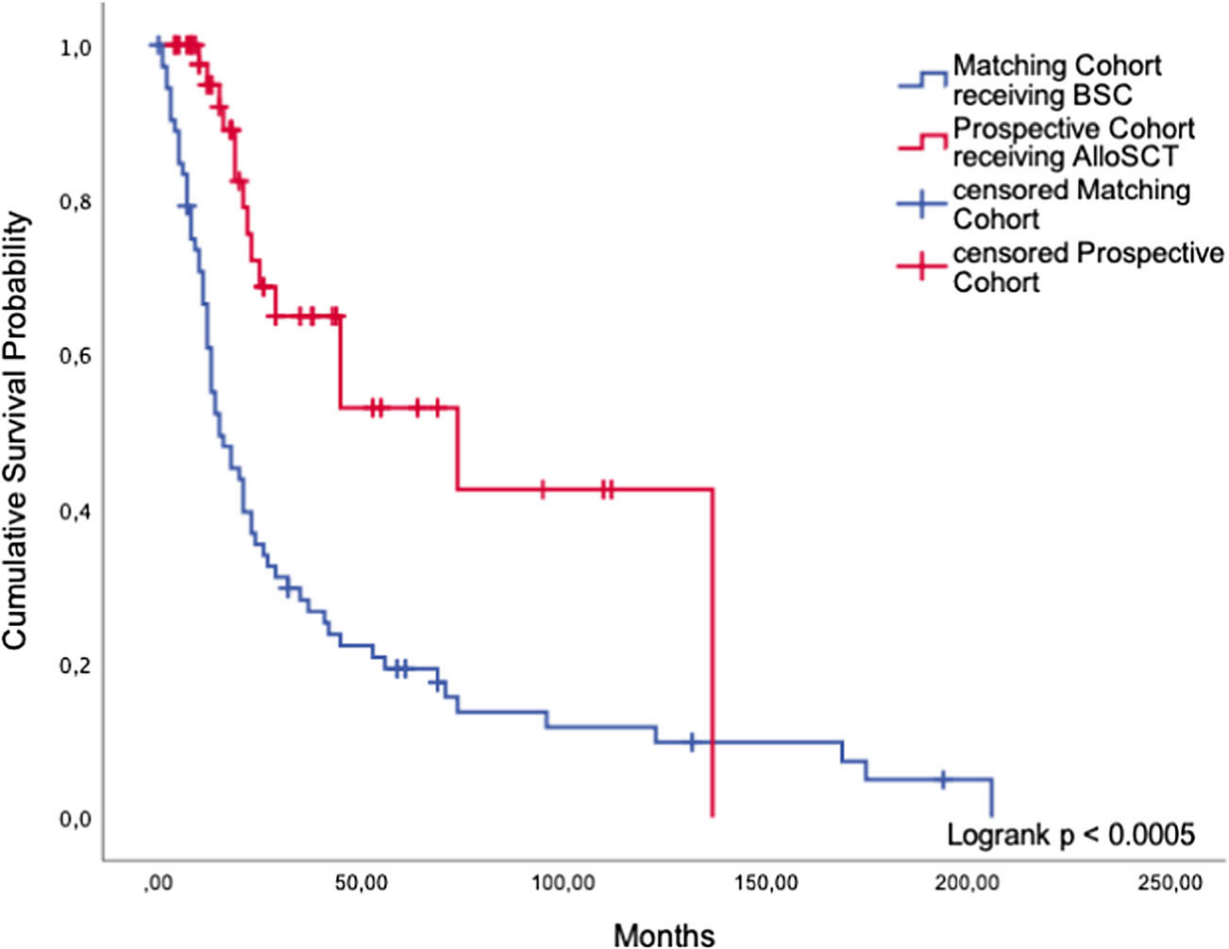
AlloSCT. Comparison of cumulative survival between patients in the prospective cohort receiving alloSCT and patients receiving BSC only

In our matched-pair analysis, 47 patients who underwent alloSCT were compared with 73 matched patients who were treated with BSC only. Transplanted patients achieved a highly significant survival benefit compared with eligible patients who did not undergo alloSCT (median OS 74 months (95%CI: 24.4; 123.6) versus 15 months (95%CI: 7.8; 22.2)) (*p* < 0.0005, Fig. 6). As we were looking at a historical matching cohort, it was not possible to ascertain why alloSCT was not carried out in the latter patients who were formally eligible according to guideline criteria.

## Discussion

Due to strict eligibility criteria, MDS patients enrolled in clinical trials are a selected subgroup not representative of the entire patient population. Therefore, real-life evidence of the survival impact of certain therapeutic approaches is sparse. The Duesseldorf MDS Registry approximates the characteristics of the general MDS population and thus provides robust information about the clinical value of widely used therapies.

When treating MDS patients, there are two major treatment goals: to improve the patients’ quality of life mainly with non-intensive measures and to prolong overall survival especially in high-risk MDS patients. However, low-risk patients treated with BSC only with a durable hematologic improvement tend to show superior overall survival as well, since one or more cytopenias are a primary reason for morbidity as well as mortality. As improved quality of live is closely associated to reduced mortality, we chose overall survival as our major endpoint. Furthermore, as the MDS CI adds prognostic information independently from the IPSS, we used those two prognostic scoring tools as the basis to classify our patients in both cohorts.

Our retrospective analysis of 1658 patients in cohort 1 indicates that patients eligible for a certain treatment did not gain a significant survival benefit compared with patients who did not receive it. The only exceptions were iron chelation therapy among the non-intensive BSC options, and alloSCT. When used according to the ELN guidelines, these two treatments conferred a significant survival advantage compared to the use of BSC only. The favorable effect of iron chelation in patients with lower-risk MDS may be attributable to the benefits of diminished iron-related oxidative stress, including amelioration of ineffective erythropoiesis in a minority of patients [9]. AlloSCT is the only curative treatment, but most MDS patients are not fit enough to be eligible. As previously shown, drugs like valproic acid, low-dose Ara-C, and antithymocyte globulin (ATG) are only palliative [10]. According to our analysis, the same is true for other therapies used these days, which can help to decrease disease-related morbidity and enhance the patients’ quality of life but fail to achieve a substantial gain in survival time.

It is therefore plausible that we failed to show a survival advantage of guideline-adherent treatment. This applies to all treatment categories examined. We found that a sizeable proportion of patients (25% on average) were indeed treated outside the guidelines. This was true for non-intensive treatment as well as alloSCT. Not being treated in strict accordance with the guidelines did not lead to inferior outcome in any of the therapeutic areas we analyzed. This observation supports the view that clinical advice should be consistent with accepted guidelines but should be free to include modifications according to patient-related risk factors, geriatric evaluation, and patient preferences.

In our prospective cohort including 381 patients seen in our outpatient clinic (cohort 2), we confirmed that adherence to guideline-based expert advice had no major impact on survival. Patients who chose to be treated differently had no inferior prognosis, as long as their treatment exceeded best supportive care. Again, the exception was alloSCT. In order to further examine the value of various therapies, we used a matched-pair procedure to compare guideline-adherent patients receiving a specific treatment with matched patients (historical controls) who received BSC only. We showed that patients following guideline-based expert advice to undergo treatment with lenalidomide, HMAs, or alloSCT did have a better prognosis than patients who would have been eligible for such treatment but received BSC only. This was true when treatment was strictly according to the guidelines, as in our analysis of cohort 1, or modified by expert advice.

Even in patients who do not derive a survival benefit from treatment with lenalidomide or HMAs, such treatment may be beneficial by improving blood counts and may even result in temporary remission. Palliative treatment should thus not be undervalued. If transfusion independency is achieved, this will greatly improve the patients’ quality of life and also decrease the risk of cardiovascular complications associated with chronic transfusion therapy in MDS [11, 12].

It is of importance that the presence of comorbidities per se was not a criterion to exclude patients from our analyses. According to the ELN guidelines, each patient in the retrospective as well as in the prospective cohort was classified to match the respective therapeutic category. Therefore, even comorbid patients were considered to receive a suitable treatment and a selection bias on this level can be ruled out. The groups receiving treatment were not younger and fitter.

Our findings do not invalidate the ELN guidelines but emphasize that proper patient management should go beyond guidelines and should involve shared decision-making. Guidelines should be considered a useful framework rather than a dogma. Unfortunately, we were often unable to ascertain the specific reasons for non-adherence to the guidelines, mainly because many patients were managed outside our tertiary referral center. Up to now, studies of interest validating guidelines in hematology are sparse. Existing studies concerning solid tumors focus on evaluating adherence to one certain type of drug. A field of special interest is early stage breast cancer. As the work about adherence to adjuvant hormonal therapy [13] suggests, patients’ non-adherence is associated with an increased risk of mortality. Nevertheless, studies like this are far from evaluating adherence to an entire guideline. The investigation of guideline use in other hematological entities such as acute myeloid leukemia or lymphoma would be worthwhile, to gain important information on the impact of adherence to patient related outcomes.

Altogether, our retrospective and prospective analyses imply that, with the exception of alloSCT, none of the currently available therapies is powerful enough to render deviation from guideline-based expert advice a major disadvantage in terms of prognosis. We clearly need better treatment options, which can really make a change when correctly applied by an MDS expert.

## Supplementary Information

ESM 1 (PDF 101 kb)

## Data Availability

The datasets generated during and/or analyzed during the current study are available from the corresponding author on reasonable request.
