# Supplementary material for: Analysis of the impact of adherence to guidelines and expert advice in patients with myelodysplastic syndromes
Source: Ann Hematol. 2020 Nov 7;100(2):455–63. doi: 10.1007/s00277-020-04325-7 (PMC7817552; doi:10.1007/s00277-020-04325-7)
Supplement: Supplementary file 1 — (PDF 101 kb) [file 277_2020_4325_MOESM1_ESM.pdf]

Table 1. Baseline characteristics. Summary of baseline characteristics in the retrospective cohort 1 and the prospective cohort 2

	Retrospective Cohort 1	Prospective Cohort 2	p-value
	n = 1658	n = 381	0.015
**median age at first diagnosis**	67 (18-99)	64 (23-88)	
**gender**	n / %	n / %	
male	1015 / 61.2	233 / 61.2	1.000
female	642 / 38.7	148 / 38.8	
**WHO 2016 subtype**	n = 1653	n = 381	
MDS SLD	93 / 5.6	9 / 2.4	
MDS RS SLD	67 / 4.0	15 / 3.9	
MDS MLD	457 / 27.6	105 / 27.6	
MDS RS MLD	110 / 6.6	31 / 8.1	
MDS EB 1	203 / 12.2	40 / 10.5	
MDS EB 2	254 / 15.3	53 / 13.9	0.306
MDS del(5q)	100 / 6.0	17 / 4.5	
CMML	164 / 9.9	43 / 11.3	
sAML	149 / 9.0	16 / 4.2	
MDS U	18 / 1.1	27 / 7.1	
RARS-T	11 / 0.7	6 / 1.6	
MDS-NOS	0 / 0	19 / 4.9	
**IPSS risk group**	n = 1658	n = 218	
low risk	395 / 23.8	121 / 35.9	
intermediate risk I	659 / 39.7	107 / 31.7	0.001
intermediate risk II	369 / 22.3	71 / 21.1	
high risk	235 / 14.2	38 / 11.3	
primary MDS	1366 / 82.4	247 / 73.1	<0.0005
therapy-related MDS	171 / 10.3	101 / 26.9	
**MDS CI**	n =1015	n = 347	
Low risk	445 / 43.8	179 / 51.6	0.125
Intermediate risk	447 / 44.1	128 / 36.9	
High risk	123 / 12.1	40 / 11.5	

Table 2. Baseline characteristics in the prospective cohort 2

	Adherent pts	Non-adherent pts	p-value
	n=257	n=103	
	n / %	n / %	
**Median age at diagnosis**	65 (23-88)	62 (31-82)	0.016
**gender**			0.177
male	95 / 37.0	57 / 55.3	
female	162 / 63.0	46 / 44.7	
**WHO 2016 subtype**			0.333
MDS SLD	4 / 1.7	3 / 2.9	
MDS RS SLD	12 / 5.0	3 / 2.9	
MDS MLD	73 / 30.2	27 / 26.2	
MDS RS MLD	21 / 8.7	9 / 8.7	
MDS EB 1	26 / 10.7	12 / 11.6	
MDS EB 2	36 / 14.9	14 / 13.6	
MDS del(5q)	11 / 4.5	6 / 5.8	
CMML	29 / 12.0	12 / 11.8	
sAML	11 / 4.5	4 / 3.9	
MDS U	16 / 6.6	10 / 9.7	
RARS-T	3 / 1.2	3 / 2.9	
**IPSS risk group**	n = 227	n = 93	0.170
low risk	92 / 40.5	26 / 28.0	
intermediate risk I	66 / 29.1	36 / 38.7	
intermedate risk II	45 / 19.8	19 / 20.4	
high risk	24 / 10.6	12 / 12.9	
**MDS CI**	n = 239	n = 92	0.242
low risk	120 / 50.2	50 / 54.3	
intermediate risk	87 / 36.4	35 / 38.0	
high risk	32 / 13.4	7 / 7.6	
**HCT CI**	n = 239	n = 88	0.354
low risk	80 / 33.5	38 / 43.2	
intermediate risk	63 / 26.4	15 / 17.0	
high risk	96 / 40.1	35 / 39.8	

Tables 3, 4, 5, 6, 7. Baseline characteristics in the retrospective cohort 1 separated by therapeutic category

The group of adherent patients includes guideline-adherent patients as well as patients, who did not receive a certain treatment since they did not prove eligible for it.
The group of non-adherent patients includes patients, who did not receive a therapy they proved eligible for and patients who, despite being eligible, did not receive the respective treatment.

Table 3. Baseline characteristics of adherent and non-adherent patients in the therapeutic group of erythropoietin

Erythropoietin	**Adherent pts** n = 73 n / %	**Non-adherent pts** n = 21 n / %	**p-value**
**gender**			0.363
male	43 / 58.9	10 / 47.6	
female	30 / 41.1	11 / 52.4	
**median age at diagnosis**	71 (41-87)	63 (19-78)	0.002
**IPSS risk group**			0.019
low risk	28 / 38.4	2 / 9.5	
intermediate risk I	45 / 61.1	19 / 90.5	
**WHO 2016 subtype**	n = 70	n = 21	0.470
MDS SLD	1 / 1.4	1 / 4.8	
MDS RS SLD	8 / 11.4	3 / 14.3	
MDS MLD	33 / 47.1	10 / 47.6	
MDS RS MLD	12 / 17.1	1 / 4.8	
MDS EB 1	4 / 5.7	4 / 19.0	
del 5q	3 / 4.3	2 / 9.5	
CMML	5 / 7.3	0	
RARS-t	4 / 5.7	0	
**MDS CI**	n = 62	n = 15	0.032
low risk	26 / 41.9	9 / 60.0	
intermediate risk	21 / 33.9	6 / 40.0	
high risk	15 / 24.2	0	

Table 4. Baseline characteristics of adherent and non-adherent patients in the therapeutic group of iron chelation

Chelation	**Adherent pts** n = 183 n / %	**Non-adherent pts** n = 44 n / %	**p-value**
**gender**			0.029
male	100 / 54.6	32 / 72.7	
female	83 / 45.4	12 / 27.3	
**median age at diagnosis**	67 (19-91)	70 (39-89)	0.108
**IPSS risk group**			0.162
low risk	73 / 39.4	15 / 34.1	
intermediate risk I	110 / 60.1	29 / 65.9	
**WHO 2016 subtype**	n = 178	n = 44	0.316
MDS SLD	17 / 9.5	3 / 6.8	
MDS RS SLD	18 / 10.1	6 / 13.6	
MDS MLD	74 / 41.6	21 / 47.7	
MDS RS MLD	18 / 10.1	3 / 6.8	
MDS EB 1	11 / 6.2	6 / 13.6	
MDS EB 2	2 / 1.1	2 / 4.6	
del 5q	22 / 12.4	2 / 4.6	
CMML	14 / 7.9	1 / 2.3	
RARS-t	2 / 1.1	0	
**MDS CI**	n = 128	n = 32	0.030
low risk	66 / 51.6	10 / 31.3	
intermediate risk	50 / 39.1	15 / 46.9	
high risk	12 / 9.3	7 / 21.8	

Table 5. Baseline characteristics of adherent and non-adherent patients in the therapeutic group of Lenalidomide

Lenalidomide	**Adherent pts** n = 27 n / %	**Non-adherent pts** n = 18 n / %	**p-value**
**gender**			0.798
male	8 / 29.6	6 / 33.3	
female	19 / 70.4	12 / 66.7	
**median age at diagnosis**	73 (32-85)	69 (39-89)	0.631
**IPSS risk group**			0.420
low risk	21 / 77.8	12 / 66.7	
intermediate risk I	6 / 22.2	6 / 33.3	
**WHO 2016 subtype**			
del 5q	27 / 100.0	18 / 100.0	1.000
**MDS CI**	n = 19	n = 17	0.370
low risk	13 / 68.4	8 / 47.1	
intermediate risk	5 / 26.3	8 / 47.0	
high risk	1 / 5.3	1 / 5.9	

Table 6. Baseline characteristics of adherent and non-adherent patients in the therapeutic group of Azacytidin

Azacytidin	**Adherent pts** n = 70 n / %	**Non-adherent pts** n = 102 n / %	**p-value**
**gender**			0.173
male	49 / 70.0	61 / 59.8	
female	21 / 30.0	41 / 40.2	
**median age at diagnosis**	69 (20-89)	65 (21-87)	
**IPSS risk group**			0.008
intermediate risk II	55 / 78.5	67 / 65.7	
high risk	15 / 21.1	35 / 34.3	
**WHO 2016 subtype**	n = 66	n = 100	0.012
MDS SLD	2 / 3.0	1 / 1.0	
MDS RS SLD	1 / 1.4	0	
MDS MLD	15 / 22.7	12 / 12.0	
MDS RS MLD	2 / 3.0	4 / 4.0	
MDS EB 1	16 / 24.2	14 / 14.0	
MDS EB 2	22 / 33.3	45 / 45.0	
CMML	3 / 4.6	7 / 7.0	
MDS U	2 / 3.0	1 / 1.0	
AML	3 / 4.6	16 / 16.0	
**MDS CI**	n = 53	n = 81	0.102
low risk	18 / 34.0	34 / 42.0	
intermediate risk	21 / 39.6	38 / 46.9	
high risk	14 / 26.4	9 / 11.1	

Table 7. Baseline characteristics of adherent and non-adherent patients in the therapeutic group of allgeneic stem cell transplantation

alloSCT	**adherents pts** n = 959 n / %	**non-adherent pts** n = 388 n / %	**p-value**
**gender**			0.542
male	571 / 59.5	238 / 61.3	
female	388 / 40.5	150 / 38.7	
**median age at diagnosis**	70 (18-91)	61 (18-73)	<0.005
**IPSS risk group**	n = 959	n = 388	<0.005
low risk	308 / 32.1	13 / 3.4	
intermediate risk I	398 / 41.5	116 / 29.9	
intermediate risk II	150 / 15.6	151 / 38.9	
high risk	103 / 10.8	108 / 27.8	
**WHO 2016 subtype**	n = 949	n = 387	<0.005
MDS SLD	74 / 7.8	10 / 2.6	
MDS RS SLD	58 / 6.1	0	
MDS MLD	343 / 36.1	70 / 18.1	

MDS RS MLD	88 / 9.3	16 / 4.1	
MDS EB 1	85 / 9.0	100 / 25.8	
MDS EB 2	124 / 13.1	106 / 27.4	
del 5q	92 / 9.7	5 / 1.3	
AML	62 / 6.5	79 / 20.4	
MDS U	15 / 1.6	0	
RARS-t	8 / 0.8	1 / 0.3	
**MDS CI**	n = 630	n = 211	0.015
low risk	268 / 42.5	104 / 49.3	
intermediate risk	291 / 46.2	88 / 41.7	
high risk	71 / 11.3	19 / 9.0	
